# Bibliometrics Analysis of the Research Status and Trends of the Association Between Depression and Insulin From 2010 to 2020

**DOI:** 10.3389/fpsyt.2021.683474

**Published:** 2021-07-22

**Authors:** Xiaohan Zou, Yuan Sun

**Affiliations:** ^1^Jilin Provincial Key Laboratory on Molecular and Chemical Genetic, The Second Hospital of Jilin University, Changchun, China; ^2^Public Computer Education and Research Center, Jilin University, Changchun, China

**Keywords:** insulin, depression, sex hormones, bibliometric analysis, citespace

## Abstract

Depression is one of the common mental illnesses. Because it is an important complication of diabetes, its association with changes in insulin levels and insulin resistance, the causative factors of diabetes, has attracted widespread attention. However, the association between insulin and depression has not been systematically studied through bibliometric and visual analysis. This study is based on 3131 publications of Web of Science to identify the current research status and research trends in this field. The results show that since 2010, the number of publications has been growing rapidly. Cooperative network analysis shows that the United States, the University of Toronto and Roger S Mcintyre are the most influential countries, research institutes and scholars, respectively. Insulin resistance, obesity, and metabolic syndrome are hot topics in this field. Analysis of keywords and references reveals that “sex hormones,” is new research area that constantly emerging. As far as we know, this study is the first one to visualize the association between depression and insulin and predict potential future research trends through bibliometric and visual analysis.

## Introduction

Depression, a common mental illness that severely reduces the quality of life, is accompanied by a high incidence of suicide. In 2008, WHO predicted that depression would rank first cause of burden of disease worldwide (1). The key molecular mechanisms associated with depression include the “monoamine hypothesis,” “hypothalamus-pituitary-adrenal (HPA) axis disorder,” “inflammation,” “neural plasticity,” “gene,” and “environment” (2). In recent years, the viewpoint on the pathogenesis of depression has been move away from only the roles described above as the primary cause of depression to more comprehensively (3). Changes in the endocrine system are also an important causes of depression pathophysiology. Research on changes of non-psychiatric symptoms link to affective disorders is increasing (4, 5). For example, heart disease occurs more frequently in depressed patients (6), and among the factors associated with depression, diabetes have received widespread attention. Studies have shown that about 30% of diabetic patients also suffer from depression (7, 8). Among diabetic patients, the incidence of depression is twice that of the general human (9), which indicates that there is a close association between the two diseases.

Diabetes, metabolic disorder caused by insufficient insulin secretion or insulin utilization disorder. Studies have found that insulin deficiency or insulin resistance can also be observed in patients with depression (10).

Insulin, a protein hormone secreted by pancreatic β cell, is the only hormone in the mammal body that could lower blood glucose, and promote the synthesis of glycogen, fat, and protein. Insulin was isolated by Frederick Grant Banting and Charles Herbert Best in 1921 (11), which is one of the most important biomedical events of the last century. Insulin is transported through the blood-brain barrier by saturated transport system, and its receptors are distributed throughout the brain (12). Insulin receptors are found in brain areas not related to food intake, indicating that insulin has an effect on other brain functions (13). For example, the down-regulation of insulin receptor expression in the hypothalamus causes depression-like behavior in rats (14).

The mechanism of depression as a complication of insulin deficiency or insulin resistance is not fully clear, however, appears to have a few routes.

There is evidence that depression and diabetes could share same symptoms, especially the misalignment of the HPA axis, could cause insulin resistance, cardiovascular disease, depression, type 2 diabetes, and increased mortality (9). Insulin signaling has been shown to regulate the dopaminergic and serotonergic systems, which are pathways that play a key role in depression. The administration of central insulin can increase the expression of dopamine transporter in rats (15, 16). On the other hand, it has also been shown that insulin could increase the level of serotonin in the brain, therefore may have a promoting effect on mood (17). Insulin reduces the activity of monoamine oxidase-A and monoamine oxidase-B, the enzymes which degrade serotonin and dopamine, thereby, increasing the activity of these systems and exhibiting antidepressant effects (18, 19).

Publications have proved the connection between insulin and depression. However, as far as we know, no one has systematically analyzed the subject of this field by means of bibliometric analysis. Therefore, study aims to comprehensively analyze current status and development trend on the link between insulin and depression from 2010 to 2020 through bibliometrics. Previous reviews only rely on individuals to study the research through literature summary and extraction, cannot fully reflect the temporal and spatial distribution of researcher, institution, and journal. Moreover, it is difficult to visualize the internal structure of the knowledge base and research focus, and rarely find systematic, comprehensive, and visual research.

Bibliometric analysis was proposed in 1969, by Pritchard, and plays an important role in reflecting the characteristic and future trend (20, 21). In recent years, the bibliometric analysis software CiteSpace has been used by scholars in medicine and biology academic research, and has attracted widespread attention for its scientific calculations and image functions (22). In this study, the Web of Science Core Collection (WOSCC) and CiteSpace have been used for bibliometric and visual analysis. Literature related to depression and insulin is collected into a specific database. CiteSpace is used to perform statistical calculations and generate visualization results for different node. This research aims to:

1. Investigate the output and growth trends of publications in the field of the association between insulin and depression.
2. Investigate the core countries/regions, institutions, authors, and journals.
3. Identify international scientific cooperation network between countries/regions, institutions, and authors.
4. Explore key themes, hotspots, and research trends.

Our research aims to fill the gaps and effectively observe the development of this field. The research results are helpful for studying the association between depression and insulin and helping follow-up researchers to determine journal publications and collaborators. Timely analysis of keywords, themes, and research trends could promote the development to the elucidation of the cause, prevention, and treatment of depression.

## Materials and Methods

### Methods

This study uses CiteSpace5.7.R4 (64-bit) to analyze existing research related to insulin and depression, and aims to provide reference, scientific, and intuitive. CiteSpace, a Java application which could perform bibliometric analysis, created by Professor Chen Chaomei (College of Computing and Informatics, Drexel University, Philadelphia, PA, USA) (23). First, a series of homogeneous networks are derived from a series of time interval slices of equal length. According to the time slice, the different settings of etymology and thresholds, different and pertinent conclusions can be drawn (24). These networks are combined and visualized in a map, identifying collaboration network. Core countries/regions, institutions, and authors and their cooperative relationships could be identified by collaboration network. Co-citation references and co-occurrence words could reveal the research foundation and hotspots. In addition, an important role of CiteSpace is the detection of burstness keywords and references. The function intuitively identifies the development trends, provides scientific and intuitive reference and auxiliary support for scholars in this direction (20). So far, it is under continuous development and is widely used by researchers in the field of biomedical research (25, 26).

### Data Sources

Bibliometric analysis bases on a specific range of database. The database is from: WOSCC. In order to avoid the prejudice caused by the database update, we conducted a literature search from the WOSCC on February 22, 2021.

The search time range is from 2010 to 2020. Insulin and depression are included in the search strategy: TS = insulin AND TS = depressions OR depression OR depressive OR depressed OR despondent.

In this study, the search criterion in the WOSCC database is as follows:

- Time frame: 2010 to 2020;
- Document type: articles and reviews;
- No species restriction;
- Language: English

**Figure 1:**
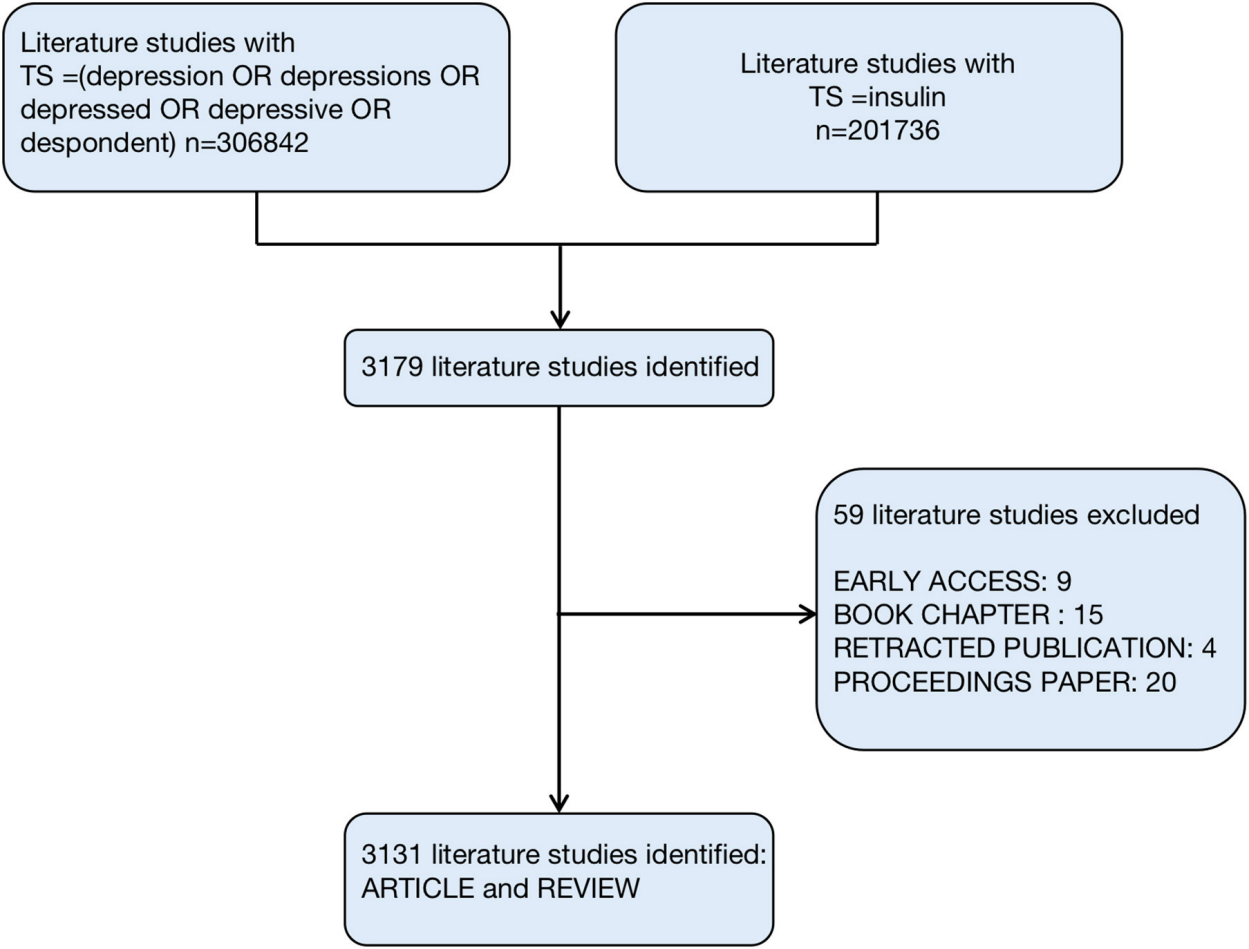
Flowchart for including and excluding literature studies.

A total of 3179 records were retrieved. Then we excluded “BOOK CHAPTER,” “RETRACTED PUBLICATION,” “EARLY ACCESS,” and “PROCEEDINGS PAPER.” Finally, 3,131 documents were determined. Figure 1 shows the steps. The search results are exported in CiteSpace for further analysis.

### Data Analysis

The analysis in this study uses WOSCC to analyze the publication output, web of science subject category, publication year, IF and other functions.

After that, the selected documents were imported into CiteSpace, and time span was 2010–2020. Select the node based on the type of analysis need to perform. The specific parameter settings in CiteSpace are as follows: Time slicing: January 2010–December 2020, Term source: Title, Abstract, Author Keywords and Keyword Plus, Node type: Author, Institution, Country, Keyword, Reference, Cited Author and Cited Journal, Link strength: Cosine, Selection Criteria: Top *N* = 50.

In the generated map, the color of the circular nodes represents the time when the article was published. The thickness of the node is positively related to the frequency, the thickness of the line describes the strength between the projects, and the color of the line describes the year when the two projects first collaborated. The nodes in the map represent elements such as author, country/region, or institution. The link lines between the nodes indicate the collaboration relationship. The larger the circle, the more articles published. The wider the line, the stronger the relationship. The outermost purple ring represents the center level (27).

After sorting out countries/regions, institutions, authors, and their relationship, researchers could find teams for potential cooperation. A highly concentrated ranking of keywords and references and research on burstness can be used to analyze research status and hotspots, and further explore to future research trends in the field of insulin and depression.

## Results

### Publication Years

**Figure 2:**
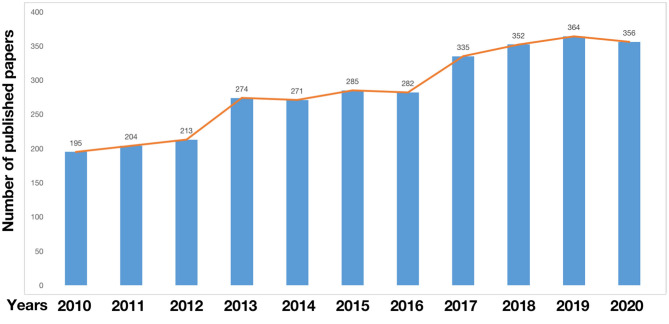
Annual trend chart of publications.

The change in the number of papers is an important indicator that can visually show the development trend of the field. From 2010 to 2020, the annual publications on insulin and depression included a total of 3,131 related articles. The distribution is shown in Figure 2.

In 2013 and 2017, publications grew rapidly. In 2017, the number of publications exceeded 300. Although, the number of publications declined in 2014, 2016, and 2020, the number of papers published each year is basically on the rise.

### Scientific Collaboration Network

Seventy-five countries or regions have contributed to publications in the field of research on the association between depression and insulin. Table 1 lists the top 10 countries or regions ranked by publication and centrality. The greater the centrality value, the more cooperation between the node, and other nodes. In order of number of publications, the top 5 countries or regions are the United States, People's Republic of China, Canada, Germany, and England. The top five countries/regions by centrality are the United States, England, Canada, Mexico, and Australia. A comprehensive analysis of centrality shows that the United States (publication: 998, centrality: 0.33), England (publication: 200, centrality: 0.20), Canada (publication: 247, centrality: 0.14), and Mexico (publication: 31, centrality: 0.11) are the most influential in this field. The nodes marked with a purple ring indicate centrality >0.1.

**Table 1:** Top 10 countries/regions, institutions, and authors in terms of publications and centrality.

**Items**	**Publication**			**Centrality**		
	**Ranking**	**Name**	**Number**	**Ranking**	**Name**	**Number**
Country/region	1	USA	998	1	USA	0.33
	2	People R China	328	2	England	0.20
	3	Canada	247	3	Canada	0.14
	4	Germany	202	4	Mexico	0.11
	5	England	200	5	Australia	0.08
	6	Australia	172	6	Italy	0.08
	7	Brazil	149	7	Egypt	0.08
	8	Italy	144	8	Germany	0.07
	9	Netherlands	135	9	Brazil	0.07
	10	Japan	110	10	Spain	0.07
Institution	1	University of Toronto	79	1	University of Toronto	0.17
	2	Harvard University	42	2	Harvard University	0.12
	3	Deakin University	41	3	Stanford University	0.09
	4	Stanford University	39	4	Penn University	0.08
	5	University of California, San Francisco	33	5	King's College London	0.06
	6	Emory University	31	6	Emory University	0.05
	7	Washington University	30	7	Washington University	0.05
	8	Monash University	28	8	University of Copenhagen	0.05
	8	University of Pittsburgh	28	9	University of Melbourne	0.05
	10	University of Copenhagen	25	10	University of Illinois	0.05
Author	1	Roger S Mcintyre	38	1	Andre F Carvalho	0.02
	2	Zatollah Asemi	20	2	Brenda W J H Penninx	0.02
	3	Elisa Brietzke	18	3	Michael Berk	0.02
	4	Rodrigo B Mansur	17	4	Roger S Mcintyre	0.01
	5	Fereshteh Bahmani	12	5	Elisa Brietzke	0.01
	6	Andre F Carvalho	11	6	Rodrigo B Mansur	0.01
	7	Jane Speight	9	7	Natalie Rasgon	0.01
	8	Mehala Subramaniapillai	9	8	Reininghaus Eva	0.01
	9	Pouwer F	9			
	10	Brenda W J H Penninx	8			

**Figure 3:**
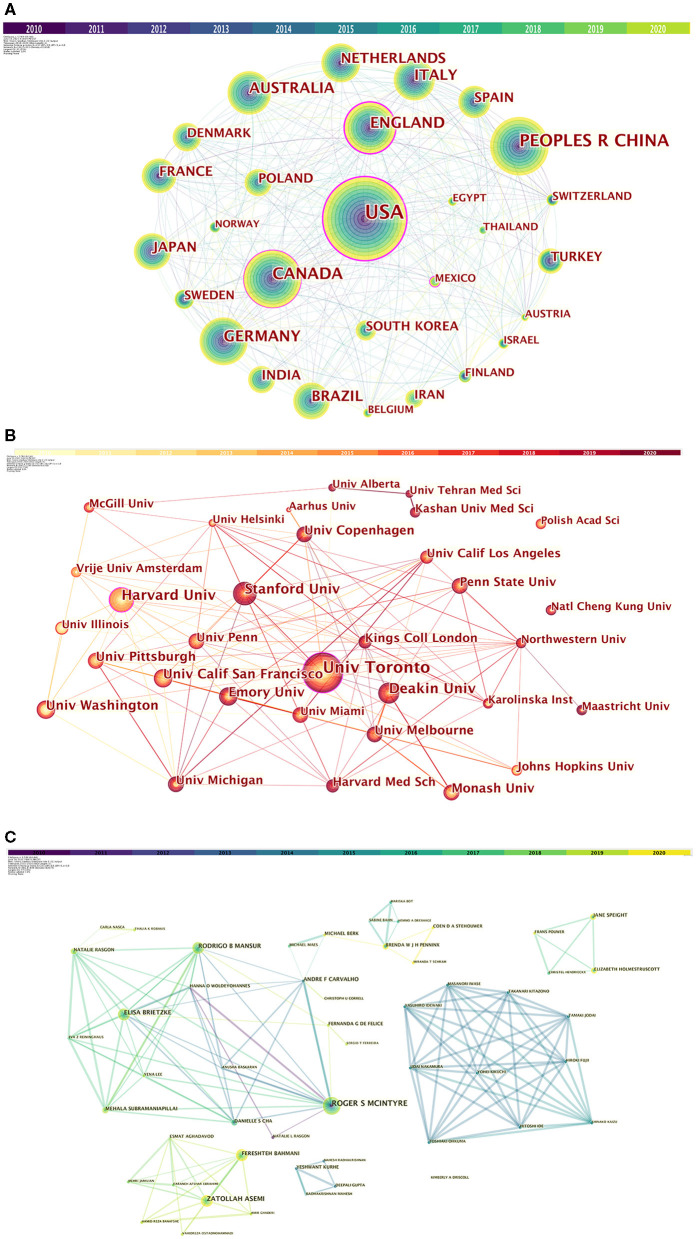
**(A)** Collaboration among countries/regions. **(B)** Collaboration among institutions. **(C)** Collaboration among authors.

The network diagram of collaboration between countries/regions is shown in Figure 3A. In this study, nodes represent more than 25 articles published, totaling 30. The larger the node, the more articles be published. The change in node color indicates the time of published. The connections between the nodes represent the cooperative relations of the countries/regions. As can be seen from the figure, there are many links between countries. The United States ranks first in the total number of articles published and centrality, and the USA connected to almost all the countries in the figure. It is worth noting that Mexico has 31 publications, ranking 24th, but centrality is 0.11, ranks fourth. It shows that although, Mexico does not publish much, it has lots of cooperation with other countries. The total number of articles published in China ranks second, but the centrality is only 0.02, reflecting China's lack of international cooperation in research. In the follow-up development, it can be expected that China will strengthen cooperation with other countries in the direction of depression.

From 2010 to 2020, a total of 3,131 institutions in this field published papers. Table 1 lists top 10 institutions based on publication and centrality. The top 5 institutions that have contributed the most in this field are: University of Toronto, Harvard University, Deakin University, Stanford University, University of California, San Francisco. The generated organization network map identified 513 nodes and 1,358 link lines, representing organizations, and their cooperative relationships. As a result, extensive cooperation between institutions was discovered. On the basis of the analyses of publications and centrality, University of Toronto (Publications: 80, Centrality: 0.15), Penn University (Publications: 25, Centrality: 0.10), Stanford University (Publications: 40, Centrality: 0.09), Harvard University (Publications: 42, Centrality: 0.07), University of Copenhagen (Publications: 27, Centrality: 0.06) were the major institutions. The generated organization network map identified 513 nodes and 1,358 link lines, representing organizations, and their cooperative relationships (Figure 3B). For example, the University of Toronto represented by the largest node has the most publications and has extensive cooperation with Stanford University, Queen's University, Harvard University, the University of Pittsburgh, and the University of California, San Francisco. Although, Penn University has few publications, it is highly central and has many research partners, such as University of California Los Angeles, University of California San Francisco, University of Alabama, Birmingham, Stanford University, etc.

Table 1 lists the top 10 authors of publications and 8 authors with centrality ≥0.01. Six authors have published more than 10 papers in this field. Among 10 authors, Roger S Mcintyre ranked first with 38 papers. His centrality is also in the first place. The co-author network is shown in Figure 3C, which contains 492 nodes and 899 connections. The link between the node and the node represents the author and the cooperative relationship. Since the centrality <0.1, no node is marked with a purple ring. As shown in the figure, the relationship between the top-ranked Roger S Mcintyre and Zatollah Asemi is restricted. The centrality of Zatollah Asemi, who is in second place, is <0.01, indicating that he has not played a central role in the cooperation. Although, there is no direct connection between the three authors whose centrality is >0.02, they have an important role in the cooperation.

### Analysis of Journals and Co-cited Journals

Articles on the association between insulin and depression have been published in 1,125 different journals. Table 2 lists the top 10 journals and co-cited journals on the relationship between insulin and depression. The journals identified in this table mainly involve endocrinology, neurology, medicine. The journal with the highest number of publications is PLoS ONE, followed by Diabetes Care and Journal Of Affective Disorders. The IF of all journals is higher than 2.0. Co-citation analysis is an important part of bibliometrics. The analysis of co-citation of journals shows the contribution of each journal to the field. In terms of the number of citations, top three cited journals are Diabetes Care (1,587), PLoS ONE (1,171), and Diabetes (1,083).

**Table 2:** The top 10 journals and co-cited journals.

**Items**	**Ranking**	**Name**	**Counts**	**IF (2020)**
Journal	1	PLoS ONE	83	2.740
	2	Diabetes Care	48	16.019
	3	Journal of Affective Disorders	45	3.892
	4	Psychoneuroendocrinology	43	4.732
	5	Diabetes Research and Clinical Practice	34	4.234
	6	Diabetic Medicine	33	3.083
	7	Physiology Behavior	26	2.826
	8	Brain Behavior and Immunity	24	6.633
	9	Journal of Dairy Science	24	5.40
	10	Scientific Reports	20	3.998
Co-cited Journal	1	Diabetes Care	1,587	16.019
	2	PLoS ONE	1,171	2.740
	3	Diabetes	1,083	7.720
	4	Diabetologia	1,060	7.518
	5	P Natl Acad Sci Usa	930	9.661
	6	J Clin Endocr Metab	899	9.80
	7	Lancet	884	60.392
	8	Jama-j Am Med Assoc	836	44.405
	9	New Engl J Med	832	66.10
	10	Biological Psychiatry	753	12.095

### Research Topic Analysis

#### Analysis of Highly Cited References

Highly cited references refer to publications that are cited a lot and have a wide range of influence. Table 3 shows the detail of the top 10 highly cited articles. The review by Ian Janssen et al. published in 2010 is the most cited article. In this review, the authors discuss the relationship between exercise and health problems such as depression, metabolic syndrome, and hypertension in school-age children and adolescents. After taking aerobic exercise, significant improvement in insulin variables were observed. Moderate exercise can also significantly improve the measurement of depression symptoms (28). The fourth-ranked article is also about the relationship between exercise, depression, and metabolic syndrome (29). The sixth and ninth publications show the effects and underlying mechanisms of insulin and insulin resistance on polycystic ovary syndrome (PCOS) and PCOS complication depression.

**Table 3:** The top 10 highly cited references.

**Items**	**Ranking**	**Cited by**	**Authors**	**Title**	**Years**
Highly cited reference	1	2,126	Janssen, Ian et al.	Systematic review of the health benefits of physical activity and fitness in school-aged children and youth.	2010
	2	1,853	Luppino, Floriana et al.	Overweight, obesity, and depression a systematic review and meta-analysis of longitudinal studies.	2010
	3	917	Katon, Wayne J et al.	Collaborative care for patients with depression and chronic illnesses.?	2010
	4	748	Booth, Frank W et al.	Lack of exercise is a major cause of chronic diseases?	2012
	5	614	Rehm, Juergen et al.	The relation between different dimensions of alcohol consumption and burden of disease: an overview	2010
	6	485	Teede H et al.	Polycystic ovary syndrome: a complex condition with psychological, reproductive, and metabolic manifestations that impacts on health across the lifespan	2010
	7	446	Duman, Ronald S et al.	Synaptic plasticity and depression: new insights from stress and rapid-acting antidepressants?	2016
	8	415	Fong, Daniel Y. T et al. (2002)	Physical activity for cancer survivors: meta-analysis of randomized controlled trials	2012
	9	367	Sirmans, Susan M et al.	Epidemiology, diagnosis, and management of polycystic ovary syndrome?	2014
	10	352	Desai, Sanjay V et al.	Long-term complications of critical care?	2011

The repetition of the theme in the literature shows that both exercise and polycystic ovary syndrome are research hotspots.

#### Analysis of Co-occurring Keywords

**Figure 4:**
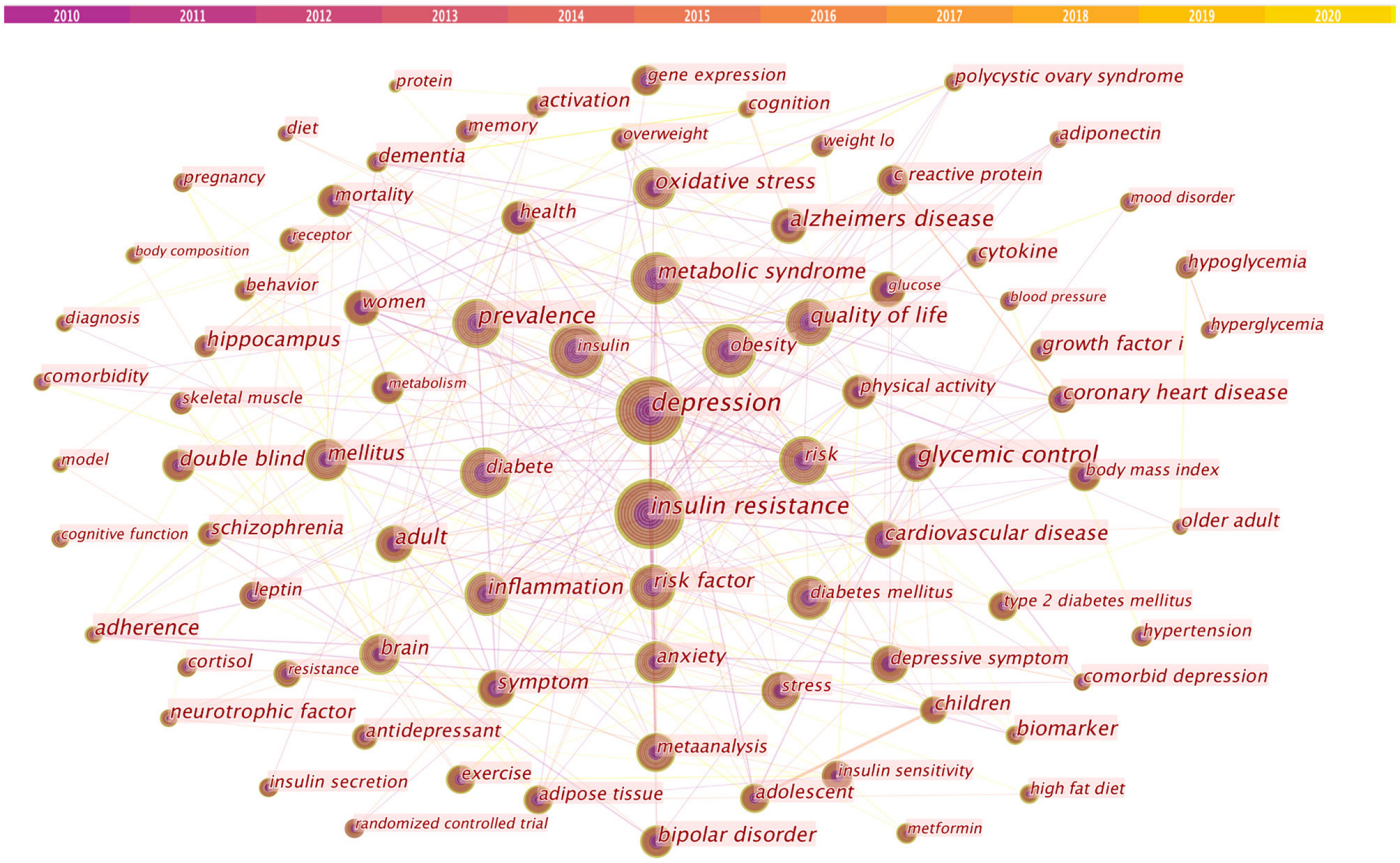
Co-occurring keywords map.

Co-occurring keywords can be used for analysis to discover research hotspots. The co-occurring keyword map of this study is shown in Figure 4. In this study, there are 561 keywords. Table 4 lists the top 20 keywords based on the frequency of co-occurring keywords. According to the co-occurrence keywords obtained from the analysis, “depression” ranks first, and it is interesting that “insulin resistance” ranks second. “Insulin” only ranks third. The fourth place is “obesity,” and the fifth place is “metabolic syndrome.”

**Table 4:** Top 20 keywords in terms of frequency.

**Ranking**	**Keyword**	**Frequency**		**Ranking**	**Keyword**	**Frequency**
1	Depression	1,004		11	Risk factor	235
2	Insulin resistance	976		12	Diabetes mellitus	235
3	Insulin	415		13	Anxiety	234
4	Obesity	389		14	Oxidative stress	221
5	Metabolic syndrome	334		15	Mellitus	204
6	Prevalence	303		16	Glycemic control	190
7	Risk	296		17	Adult	188
8	Diabetes	269		18	Brain	180
9	Quality of life	268		19	Cardiovascular disease	177
10	Inflammation	255		20	Stress	175

As shown in Table 5, cluster analysis of co-occurring keywords using CiteSpace reveals the main themes. In general, when the silhouette >0.7, the cluster is highly efficient and convincing, when the silhouette >0.5, the cluster is reasonable. We obtained five clusters with the silhouette >0.5 and three clusters silhouette >0.7. According to the cluster analysis of keywords, the five clusters are “Diabetes mellitus,” “Insulin-like grown factor,” “Metabolic syndrome,” “Depressive symptom,” and “Polycystic ovary syndrome.”

**Table 5:** Keywords cluster analysis (the silhouette value is over 0.5).

**Cluster ID**	**Size**	**Silhouette**	**Label (LLR)**	**Mean year**
0	141	0.787	Diabetes mellitus	2012
1	139	0.666	Insulin-like grown factor	2013
2	107	0.716	Metabolic syndrome	2013
3	106	0.696	Depressive symptom	2014
4	78	0.714	Polycystic ovary syndrome	2013

According to Co-occurring keywords and clusters, insulin resistance, diabetes, and metabolic syndrome are all hot research topics. The density of insulin receptors in the amygdala and hippocampus is high, and insulin resistance is highly correlated with cognitive and learning deficits (30). The early symptoms of depression increase insulin resistance, which is not related to obesity, and insulin resistance is a risk factor for the deterioration of existing depression symptoms (31, 32). Inflammation is the main pathophysiological link between depression and metabolic syndrome. The two diseases are characterized by the increase of pro-inflammatory cytokines, leptin, and insulin and glucocorticoid receptor resistance. Glucocorticoids have acute anti-inflammatory effects, but in the case of depression, glucocorticoid receptors become insensitive. In chronic depression and metabolic syndrome, both glucocorticoids and insulin receptors are in a desensitized state (33, 34).

### Future Research Direction Analysis

**Figure 5:**
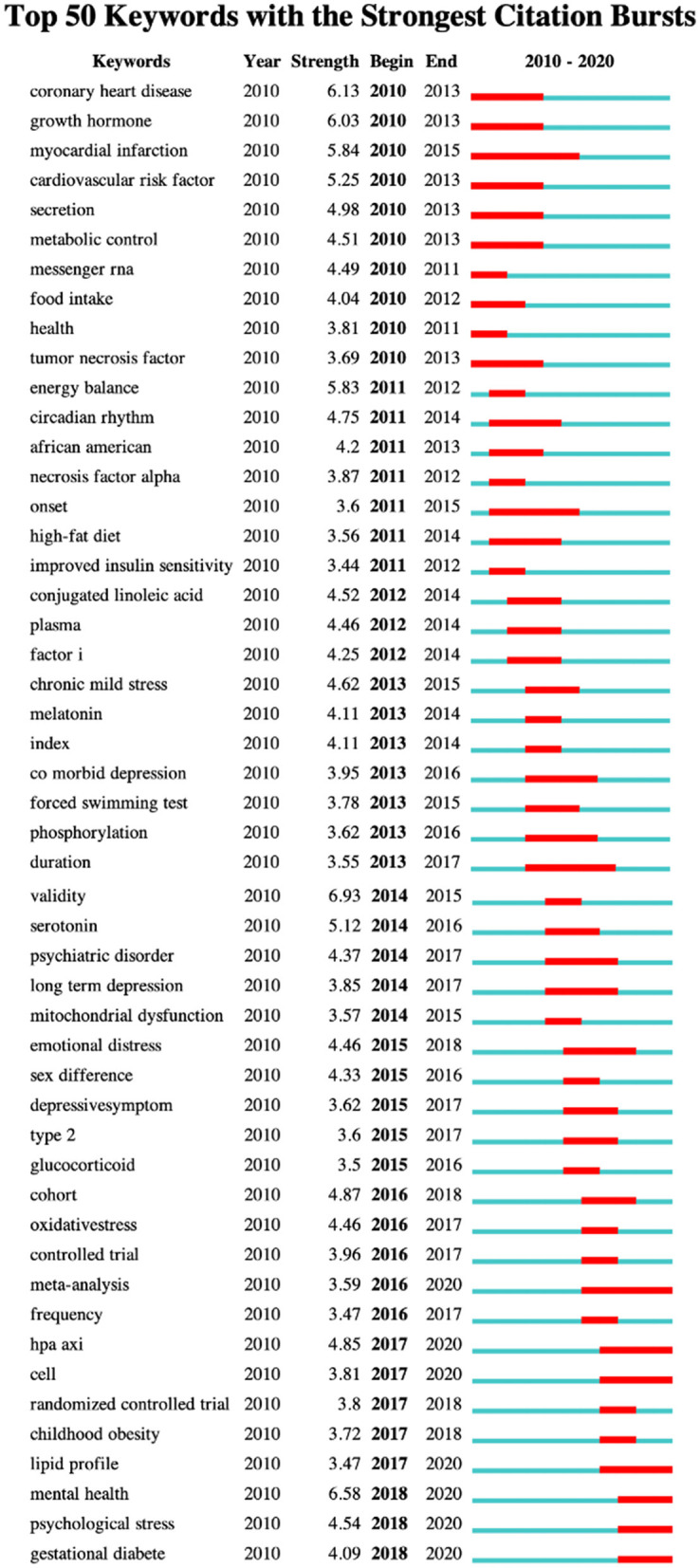
Top 50 keywords with strongest citation bursts.

Keyword burstness detection can track hot topics and research trends. The blue line represents the time interval, and the red line represents the duration of the citation outbreak, shows the evolution of hot topics. Figure 5 lists the top 50 keywords of strong citation bursts.

Hot topics from myocardial infarction, coronary heart disease, growth hormone, energy balance, and so on. Turn to bariatric surgery, oxidativestress, glucose homeostasis, lipid profile, psychological stress, gestational diabete. Keywords that last until 2020 include: meta-analysis, hap axi, cell, lipid profile, psychological stress, gestational diabete, which reflect the latest research trends.

## Discussion

According to the results of Table 3, the first and fourth publications are all about exercises. The two publications are not directly discussing the association between insulin and depression, but use the form of a review to summarize the relationship between exercise and depression, and the relationship between exercise and metabolic syndrome represented by abnormal insulin levels. In experiments with MDD patients, the experimenters found that the effect of physical exercise on the patients is comparable to the effect of standard antidepressant drugs (sertraline). Both are better than placebo for MDD patients (35). Insulin and exercise have a share mechanism to relieve depression (30, 36).

Experiments have shown that the relief of depression caused by exercise is related to the regulation of HPA axis homeostasis by exercise (37, 38) and its influence on neurotransmitter (39–41). In the brains of intensively running rats, the concentration of dopamine was significantly increased, and the density of dopamine receptors was significantly decreased, resulting produce a co mpensatory down-regulation of dopaminergic receptor sites (42). Exercise could significantly increase the level of hippocampal serotonin (5-HT) and reduce the level of 5-HT transporter (5-HTT), thereby producing antidepressant and anti-anxiety effects (43, 44).

Insulin can also increase the speed of neuronal serotonin synthesis (45). Insulin stimulates the cis-regulatory elements associated with the promoter of the enzyme tyrosine hydroxylase (TH), which is involved in dopamine synthesis, increasing the level of TH mRNA, and increasing the expression of TH gene (46).

Although, there is no article explaining the direct relationship between exercise, depression, and insulin. Based on the common mechanism of the effect of insulin on depression and the effect of exercise on depression, it can be speculated that the positive effect of exercise on metabolic homeostasis may be due to the recovery of insulin levels in the body. These publications provide a therapeutic target for future research and the concurrent of diabetic and depression.

The explosion of burstness keywords can also be used to analyze the development of research. As the Figure 5 shows, gender-based research has always been a hot research topic. From 2015–2016 “sex differences” to 2018–2020 “gestational diabetes.” Although, the research on the cardiovascular system is more intense and has more burstness keywords, such as myocardial infarction 2010–2015, coronary heart disease 2010–2013, cardiovascular risk factor 2010–2013, there are no hot spots in this field after 2010–2015. This reflects the trend of research.

On the other hand, according to the results of Table 3, the sixth and ninth publications show the effects and underlying mechanisms of insulin and deprssion on PCOS, a complex endocrine and metabolic disorder common to women of childbearing age, characterized by chronic anovulation (disorder or loss of ovulation), and hyperandrogenemia (excessive production of male hormones in women).

Although, the diagnosis of PCOS does not require insulin level testing, it is clear that abnormal insulin levels and insulin resistance play an important role in the pathogenesis of PCOS (47, 48). The incidence of insulin resistance in PCOS is between 50 and 70% (49, 50), and has nothing to do with obesity (51). Women with PCOS have an increased risk of many mental illnesses. 35% of PCOS patients have depression, compared with 10.7% in the control group (*P* < 0.0001) (52). Although, the exact mechanism that causes the increase in depression and anxiety symptoms in women with PCOS is unclear, many underlying factors may play a role.

Hyperinsulinemia caused by insulin resistance acts on gonadotropins together with luteinizing hormone (LH) increases androgen production in follicular membrane cells and reduces the production of sex hormone binding globulin in the liver, leading to the body's production of a hyperandrogen state (53, 54). Rapid hormonal fluctuations, especially functional hyperandrogen, is related to the increase in the prevalence of depression in women. Testosterone has been shown to significantly increase serotonin transporter binding, thus it is revealed that testosterone can exhaust synaptic serotonin by increasing the reuptake capacity of neurons, and ultimately trigger depression (55–57). In addition, elevated testosterone levels and hypercortisolemia have been observed in women suffering from severe depression, indicating that the HPA axis is overactive, which is related to the synthesis and activity of gonadal steroid hormones. However, not all studies have shown a consistent association between female depression and testosterone levels (58–60). Part of the reason for these inconsistencies may be due to the different designs of these studies and the classification of women according to menopausal status. These research results provide strong support for research in this field. This is not only reflected in the gestational diabete in the burstness keyword. In the highly cited literature, PCOS is also a focus. Whether in the menopausal period or in the PCOS patient population, the changes of sex hormones are the key factors that affect the physiological state. Estrogen can reduce insulin resistance in estrogen-deficient rats (61). 17β-estradiol (E2) can directly regulate the action of insulin through its action on insulin-sensitive tissues, and it can also regulate the action of insulin indirectly by regulating factors such as oxidative stress (62, 63). E2 can also mediate its protective effect on insulin by reducing inflammation (64). Currently, in individuals with MDD, the reduction in hippocampal volume is a consistent finding. In rat experiments, estrogen and progesterone activate N-methyl-d-aspartate (NMDA) receptors and participate in regulating synapse formation. Estrogen may affect mood through its influence on the plasticity of hippocampal neurons. And estrogen receptor antagonists can prevent this effect (65). Estradiol can enhance the antidepressant effect of selective serotonin reuptake inhibitors in women. After female menopause, the reactivity of serotonin decreases. The use of estradiol restored the serotonin reactivity (66–68). However, this relationship is not consistent in conditions of high E2 levels such as polycystic ovary disease, obesity, or pregnancy (69). This difference may take into account the dose and comorbidities. At present, the research of sex hormones in depression with diabetes is still in its infancy. Because it affects both insulin and depression, this may be a target for future research.

This study has some limitations. First, our research selected the core database of Web of Science and only selected English articles, which may lead to incomplete research. Second, the quality of articles is not completely guaranteed, and the impact factor is not limited. These reasons may lead to certain deviations in the research results. Third, this article cannot ensure that every publication is fully related to the subject that meets the search criteria. However, we have collected enough publications to reflect the overall state of the field.

## Conclusion

This study is based on bibliometric analysis and systematically evaluated the association between insulin and depression through 3,131 publications of Web of Science from 2010 to 2020. According to the analysis of the publications and references, it could be found that the study of shared mechanisms between depression and metabolic diseases caused by changes in insulin levels or insulin resistance has attracted widespread attention. Both the United States and China have made significant contributions to the number of publications. Research institutions in the United States, England, and Canada have contributed to the centrality of publications. Combining the burstness analysis of keywords and references, possible directions for future research could be determined. Firstly, future research needs to be no longer limited to the study of the effects of insulin on a single disease, but to link diseases with shared mechanisms to explore in-depth molecular mechanisms and find new directions for disease treatment, such as depression, diabetes, polycystic ovary syndrome. In addition, research on insulin and depression is no longer limited to traditional perspectives such as nutrient metabolism, monoamine neurotransmitters, and HPA axis. Research of the effects of changes of sex hormone and insulin levels in female perinatal and menopausal on depression will increase. This timely review analyzes the results of the association between insulin and depression may promote the development of this field and laying foundation for future research.

## Author Contributions

XZ wrote the first draft of the manuscript. YS provided the critical revisions. Both authors revised the manuscript and approved the submitted version.

## Conflict of Interest

The authors declare that the research was conducted in the absence of any commercial or financial relationships that could be construed as a potential conflict of interest.
